# Exploring the Effects of Mitonuclear Interactions on Mitochondrial DNA Gene Expression in Humans

**DOI:** 10.3389/fgene.2022.797129

**Published:** 2022-06-29

**Authors:** Edmundo Torres-Gonzalez, Kateryna D. Makova

**Affiliations:** Department of Biology, The Pennsylvania State University, University Park, PA, United States

**Keywords:** gene expression, genetic ancestry, mitonuclear DNA discordance, mitonuclear incompatibility, mitochondrial DNA, mitonuclear coevolution

## Abstract

Most mitochondrial protein complexes include both nuclear and mitochondrial gene products, which coevolved to work together. This coevolution can be disrupted due to disparity in genetic ancestry between the nuclear and mitochondrial genomes in recently admixed populations. Such mitonuclear DNA discordance might result in phenotypic effects. Several nuclear-encoded proteins regulate expression of mitochondrial DNA (mtDNA) genes. We hypothesized that mitonuclear DNA discordance affects expression of genes encoded by mtDNA. To test this, we utilized the data from the GTEx project, which contains expression levels for ∼100 African Americans and >600 European Americans. The varying proportion of African and European ancestry in recently admixed African Americans provides a range of mitonuclear discordance values, which can be correlated with mtDNA gene expression levels (adjusted for age and ischemic time). In contrast, European Americans did not undergo recent admixture. We demonstrated that, for most mtDNA protein-coding genes, expression levels in energetically-demanding tissues were lower in African Americans than in European Americans. Furthermore, gene expression levels were lower in individuals with higher mitonuclear discordance, independent of population. Moreover, we found a negative correlation between mtDNA gene expression and mitonuclear discordance. In African Americans, the average value of African ancestry was higher for nuclear-encoded mitochondrial than non-mitochondrial genes, facilitating a match in ancestry with the mtDNA and more optimal interactions. These results represent an example of a phenotypic effect of mitonuclear discordance on human admixed populations, and have potential biomedical applications.

## Introduction

Two distinct genomes—nuclear DNA (nuDNA) and mitochondrial DNA (mtDNA)—encode mitochondrial protein subunits that must act in concert to achieve optimal energy production, fatty acid synthesis, and response to stress (Kastaniotis et al., 2017). This is particularly important for tissues that are energetically demanding, such as muscle and nervous tissues. Over a 1,000 of mitochondrial proteins are encoded in the nucleus (Pagliarini et al., 2008; Sloan et al., 2015), whereas only 13 are encoded in the mitochondrial genome. mtDNA proteins contribute to forming complexes of the electron transport chain, the site of ATP production during oxidative phosphorylation (Zhao et al., 2019). Complex I, Complex III, Complex IV, and Complex V contain the mtDNA genes *ND1-6, CYB, CO1-3,* and *ATP6* together with *ATP8*, respectively (Cuperfain et al., 2018); Complex II is exclusively formed from nuDNA-encoded proteins. The nuDNA- and mtDNA-encoded proteins must physically interact with each other to form complexes in the electron transport chain, but must also interact with mtDNA during its replication and transcription (Bar-Yaacov et al., 2012; Levin et al., 2014; Hill, 2020). As a result, mtDNA and nuDNA are expected to co-evolve, a phenomenon termed *mitonuclear coevolution* **,** and have to compensate for a higher mutation rate at mtDNA than at nuDNA (Levin et al., 2014). Hence, a disparity in genetic ancestry between these two genomes (termed *mitonuclear DNA discordance*), as observed in recently admixed populations, could lead to impaired mitochondrial function, i.e., to some level of *mitonuclear incompatibility*.

Mitonuclear coevolution has been observed in a variety of eukaryotes, including yeast (Wolters et al., 2018), copepods (Ellison and Burton, 2008; Ellison and Burton, 2010; Barreto et al., 2018; Pereira et al., 2021), fruit flies (Mossman et al., 2016; Mossman et al., 2019; Camus et al., 2020), fish (Larmuseau et al., 2010), and birds (Wang et al., 2021). Studies in natural populations often demonstrated a biogeographical pattern of mitonuclear DNA discordance, in particular in hybrid zones between populations or closely related species, with sex-biased asymmetries and adaptive introgression potentially driving discordance between genomes [reviewed in (Toews and Brelsford, 2012)]. However, there is limited evidence of mitonuclear coevolution in humans. Mitonuclear linkage disequilibrium in humans was shown to be weak (Sloan et al., 2015). Strong mitonuclear incompatibility in humans is not expected because of low levels of genetic differentiation among human populations (Sloan et al., 2015; Eyre-Walker, 2017). Nevertheless, this effect is important to evaluate given its potential implications for mitochondrial replacement therapy (Gemmell and Wolff, 2015; Eyre-Walker, 2017) and for admixed populations.

Previous work (Zaidi and Makova, 2019) suggested that mitonuclear DNA discordance in admixed human populations affects mtDNA copy number, because origins of replication are encoded by mtDNA, whereas proteins important for mtDNA replication are encoded by nuDNA. MtDNA copy number, a proxy phenotype for mitochondrial function, was shown to be significantly lower at higher levels of mitonuclear DNA discordance. It was also demonstrated that local ancestry at nuDNA-encoded mitochondrial genes matches mtDNA ancestry at higher rates than expected from global ancestry estimates in African American (ASW) and Puerto Rican (PUR) populations from the 1,000 Genomes Project dataset (1000 Genomes Project Consortium et al., 2015). This observation suggests that selection acts against mitonuclear DNA discordance (Zaidi and Makova 2019).

In addition to replication, transcription of mtDNA genes might also be affected by mitonuclear DNA discordance. In humans, gene expression in the mitochondria [reviewed in (Barshad et al., 2018)] is led by a mitochondrial RNA polymerase (POLRMT), with the essential participation of the mitochondrial transcription initiation factors TFAM and TFB2M, as well as the mitochondrial transcription elongation factor TEFM. All these proteins are nuDNA-encoded. The proteins that initiate transcription agglomerate at the mtDNA promoters—LSP, HSP1, and HSP2. The promoters are separated by an hypervariable site where transcription initiation factor TFAM binds (Uchida et al., 2017). Transcription terminates with the participation of the nuDNA-encoded termination factors mTERF1-mTERF4 binding to mtDNA-encoded termination-associated sequences TAS1 and TAS2 (Barshad et al., 2018). Transcription of mtDNA results in polycistronic transcripts from the light and heavy strands. The light strand encodes the majority of protein-coding mRNAs and 14 tRNAs. The heavy strand encodes a single protein-coding mRNA (*ND6*) and eight mitochondrial tRNAs (Anderson et al., 1981; Temperley et al., 2010). Each polycistronic transcript undergoes endonucleolytic cleavage, followed by polyadenylation (D’Souza and Minczuk, 2018) in most of the mRNAs (D’Souza and Minczuk, 2018). Post-transcriptional modifications are critical for regulating translation (Pearce et al., 2017; Richter et al., 2018). The relative abundances of individual gene transcripts are ultimately also influenced by RNA degradation (Barchiesi and Vascotto, 2019), which is regulated by the nuDNA-encoded SLIRP-LRPPRC complex (Slomovic et al., 2005; Wolf and Mootha, 2014). This interplay of nuDNA-encoded transcriptional and post-transcriptional machinery acting on mtDNA sites may result in mitonuclear incompatibility, especially when there is a discordance in the genetic ancestry between the two genomes.

Mitonuclear DNA discordance has been shown to affect mtDNA transcription in copepods (Ellison and Burton, 2008; Ellison and Burton, 2010) and nuDNA transcription in fruit flies (Mossman et al., 2016; Mossman et al., 2019), particularly in response to stress. In humans, the relative abundances of mtDNA transcripts have been shown to vary widely across cell types and individuals (Ali et al., 2019). Further, allele-specific nuclear expression has been tied to genetic ancestry (Gay et al., 2020). Therefore, we expect human mtDNA gene expression to be affected by mitonuclear DNA discordance, however, this has not been previously tested.

Admixed populations are expected to have a higher level of mitonuclear DNA discordance than non-admixed populations. African Americans, a population with recent admixture, are represented in the Genotype-Tissue Expression (GTEx) dataset (GTEx Consortium, 2020), a gene expression collection of 54 tissues in approximately 1,000 individuals. Autosomal ancestry fractions in African Americans indicate mostly African ancestry (73–82%) with a smaller contribution of European ancestry [18–24%; summarized in (Goldberg and Rosenberg, 2015)]. Their mitochondrial ancestry is primarily African, whereas almost a third of Y chromosome haplotypes are of European origin (Lind et al., 2007; Stefflova et al., 2009). It was recently demonstrated (Goldberg and Rosenberg, 2015) that African Americans experienced a sex-biased admixture, likely due to a male-biased European contribution though not necessarily through a female-biased African contribution. Nevertheless, sex-biased admixture is expected to lead to mitonuclear DNA discordance in African Americans due to a substantial fraction of European ancestry in nuDNA and a majority African ancestry in mtDNA.

Here we estimated global and local genetic ancestry and analyzed mtDNA gene expression for African Americans and European Americans (a population that did not undergo a recent admixture) available in the GTEx dataset (GTEx Consortium, 2020). We hypothesized that high mitonuclear DNA discordance would result in impaired expression of genes encoded by mtDNA. Specifically, we addressed the following questions: (1) Are the levels of mtDNA gene expression different between populations with relatively high vs. relatively low mitonuclear DNA discordance? (2) Are the levels of mtDNA gene expression lower in individuals with higher mitonuclear DNA discordance, independent of the population they belong to? (3) Is there a negative correlation between mitonuclear DNA discordance and mtDNA gene expression levels? (4) Is there evidence of selection towards a matching ancestry between mtDNA and nuDNA-encoded mitochondrial genes? Our results point towards mitonuclear DNA effects on mtDNA expression, but uncover a nuanced, gene-specific picture. They also highlight the need of including a larger number of, and larger sample sizes for, diverse populations in human genetic research.

## Materials and Methods

### Gene Expression and WGS Data

The counts of normalized (in Transcripts per Million, or TPM) RNA-Seq reads mapping to protein-coding mtDNA genes were obtained from https://www.gtexportal.org/home/datasets. These were processed as specified in the GTEx data release for version 8 (GTEx Portal Documentation). The dataset contains 838 genotyped individuals for 54 distinct tissues. In our analysis, we utilized the data for genotyped individuals who self-reported as European Americans (*n* = 688) or African Americans (*n* = 101; Table 1, Supplementary Table S1). We focused on tissues that were energetically demanding and had a large sample size. Many brain subtissues were sampled in GTEx, but all of them had small sample sizes. Pooling these is unfeasible due to the high variability across subtissues. Tibial artery has a large muscular component, hence we describe it as smooth muscle (tibial artery) in our analyses. However, for esophagus (smooth muscle) the muscularis tissue was specifically dissected for each sample.

**TABLE 1:** MtDNA haplogroup designations for genotyped African Americans and European Americans utilized in the study. The haplogroups are listed in the order of decreasing numbers in the overall dataset. In parentheses is the percentage from the total number of each population. We excluded 29 individuals with non-African or non-Eurasian haplogroups.

Haplogroup/Population	mtDNA ancestry designation	African Americans (*n* = 101)	European Americans (*n* = 688)
L	African	88 (87.1%)	6 (0.9%)
H	European/Eurasian	9 (8.9%)	347 (50.4%)
U	European/Eurasian	3 (3.0%)	98 (14.2%)
T	European/Eurasian	1 (1.0%)	65 (9.4%)
K	European/Eurasian		61 (8.9%)
J	European/Eurasian		50 (7.3%)
V	European/Eurasian		22 (3.2%)
I	European/Eurasian		13 (1.9%)
X	European/Eurasian		13 (1.9%)
W	European/Eurasian		13 (1.9%)

The GTEx sequencing libraries were prepared using the Illumina TruSeq library construction protocol, which is non-stranded and enriched for poly-A tails (GTEx Consortium, 2017). Therefore we were unable to adequately study transcripts from non-protein-coding genes in mtDNA. Additionally, we excluded two protein-coding genes in mtDNA, *ND5* and *ND6,* for the following reasons. Because the RNA-Seq data are not strand-specific, we cannot definitively distinguish between *ND5* and *ND6* transcripts. *ND5* overlaps with the 3’ UTR of *ND6*, encoded on the opposite strand. The enrichment for polyadenylation also complicates studying *ND5* and *ND6*. *ND6* transcripts are not polyadenylated (Temperley et al., 2003; Temperley et al., 2010; Mercer et al., 2011; Kuznetsova et al., 2017) and even poly-A tails in *ND5* transcripts may just range from 0–10 bps (Temperley et al., 2010).

The mitochondrial genome encodes two pairs of genes as bicistronic transcripts: *ATP8/ATP6* and *ND4L/ND4*. The GTEx RNA-Seq pipeline separates genes into *ATP6*, *ATP8*, *ND4L* and *ND4*, by discarding the bases that overlap between genes (35 bp for the *ATP6/ATP8* overlap and 6 bp for the *ND4L* and *ND4* overlap). Although these genes are commonly treated separately (Lunnon et al., 2017; Ali et al., 2019; Yang et al., 2021), mapping artifacts could hinder differentiating individual genes in each bicistronic transcript. In a separate analysis, we modified the GTEx RNA-Seq pipeline (https://github.com/broadinstitute/gtex-pipeline/tree/master/rnaseq) to generate counts for the bicistronic transcripts, instead of treating them individually. Due to computational requirements of the RNA-Seq pipeline, only a subset of samples for one tissue was used (skeletal muscle, N = 157) instead of the entire GTEx dataset (789 individuals over seven tissues).

Whole-genome sequencing (WGS) data, derived from whole blood (GTEx Consortium, 2017), were utilized for the analyses of global ancestry, mtDNA haplogroup determination, and local ancestry.

### Adjusted Gene Expression

Age (ranging from 20 to 70 years) and ischemic time of the tissues were significantly correlated with normalized gene expression (as measured with Pearson correlation). Therefore, we built multiple linear regression models with these two variables for each gene and tissue. We utilized the residuals resulting from these models as “adjusted gene expression levels.” Mitochondrial DNA copy number, computed according to the method described in (Yang et al., 2021), derived from whole blood (whole genome sequencing data are unavailable for other tissues), was not significantly correlated (as measured with Pearson correlation) with mtDNA gene expression in this tissue. The expression levels were not significantly different between males and females, except for *CO2* and *ND4* in esophageal smooth muscle (Mood’s median test: *p* = 0.011 and *p* = 0.011, respectively, Supplementary Table S2). Therefore, we did not adjust for sex and for mtDNA copy number in the subsequent analyses.

### Global Ancestry

Global ancestry proportions are an average of ancestral population designations for informative SNPs across the nuclear genome. Estimates were obtained by performing a supervised analysis using ADMIXTURE version 1.3 (Alexander and Lange, 2011), assuming two ancestral populations (K = 2). Utilizing the cross-validation procedure in ADMIXTURE (Alexander and Lange, 2011) at K = 1,2,3,4,5 (Supplementary Figure S1), K = 2 was determined to be a sensible fit for our data. The 1000 Genomes Northern Europeans from Utah (CEU) and Yoruban (YRI) populations were utilized as reference populations for each ancestry fraction.

### mtDNA Haplogroup Determination

We utilized HaploGrep 2 (Weissensteiner et al., 2016) to classify the mtDNA sequences into haplogroups. Major haplogroups were then grouped into African (L) and European/Eurasian (H, I, J, K, T, U, V, W, and X) corresponding to pre-colonization origins. Some of the haplogroups in our dataset are likely of the Eurasian origin, but we considered them to be European/Eurasian for simplicity. A total of 29 genotyped samples (2 African Americans, 27 European Americans) had a non-Eurasian, non-African mitochondrial haplogroup (A, B, and C: Native American, and M, N, R, F, and Z: Asian) and were excluded from our analysis.

### Mitonuclear Discordance

Mitonuclear DNA discordance is the fraction of the nuclear genetic ancestry that does not match the mtDNA ancestry. In an individual with two ancestral fractions, X and Y, with a mitochondrial haplogroup of X ancestry, mitonuclear DNA discordance is equal to the fraction of Y nuclear ancestry. In our dataset, we have four different scenarios: an African American individual with African mtDNA ancestry, an African American individual with European mtDNA ancestry, a European American individual with African mtDNA ancestry, and a European American individual with European ancestry. In the first case (an African American individual with African mtDNA ancestry), we used the European component of nuclear ancestry estimates (subtracted the fraction of African nuclear ancestry from 100%) as a measure of mitonuclear discordance in this particular individual. In the second case (an African American individual with European mtDNA ancestry), we used the African ancestry component of nuclear ancestry estimates as the measure of mitonuclear DNA discordance. In the third case (a European American individual with African mtDNA ancestry), we used the European ancestry component of nuclear ancestry estimates as the measure of mitonuclear DNA discordance. Finally, in the fourth case (a European American individual with European ancestry), we used the African ancestry component of nuclear ancestry estimates as the measure of mitonuclear DNA discordance.

### Statistical Test for Expression Boxplots

A one-tailed Mann-Whitney U test [using the Pingouin python library (Vallat, 2018)] was performed to compare the distributions of adjusted mtDNA gene expression between European Americans and African Americans. This non-parametric test compares mean ranks between two distributions, with the null hypothesis that the distribution from one sample is equivalent to the distribution from the other sample. We chose a one-tailed test to evaluate a hypothesis that gene expression is lower in the higher discordance group. We accounted for multiple hypothesis testing with Bonferroni correction (accounts for testing in 11 genes for each tissue). Common-language effect sizes (ranging from 0 to 1, or 0%–100%) from this test indicate the probability that a randomly sampled data point from one distribution would be higher than a sampling from a second distribution. Specifically for our hypothesis, this test considers the probability that a random sample from the European American distribution would be higher than a random sample from the African American distribution of adjusted gene expression.

### Permutation Test

A permutation test was utilized to compare mtDNA gene expression and mitonuclear DNA discordance in a non-parametric manner. Our specific hypothesis here was that mean adjusted mtDNA gene expression is lower in the higher mitonuclear DNA discordance group. For each tissue and mtDNA gene, we calculated the difference in adjusted gene expression between the lower and the higher mitonuclear DNA discordance groups (with permuted labels). The mean mitonuclear DNA discordance was utilized as a cutoff value for low and high discordance groups. This cutoff value varies by tissue because of the differences in samples available per tissue. We plotted the distribution of differences in mean adjusted gene expression for 1,000 permutations which is compared to the observed expression difference. The *p*-value was computed as the number of permutations with an expression difference above the observed difference, divided by the total number of permutations.

### Correlation Test

A one-sided, Bonferroni-corrected Spearman’s rank-order correlation test was used to investigate the relationship between mitonuclear DNA discordance to mtDNA gene expression. This non-parametric test was chosen because mitonuclear DNA discordance was not normally distributed. Furthermore, a one-sided test was used to evaluate a hypothesis that there is lower gene expression with higher mitonuclear discordance.

### Local Ancestry Estimation and Enrichment

Local ancestry proportions, similar to global ancestry, are ancestral population designations for informative SNPs along the nuclear genome. However, they are more detailed by offering information for segments along each chromosome instead of a global average. Estimates were generated using RFMix (Maples et al., 2013). As a quality control step, we compared global ancestry computed from local ancestry estimates (RFMix) to global ancestry estimates from ADMIXTURE (Supplementary Figure S2). These estimates were highly correlated.

Post-admixture selection against mitonuclear discordance would result in an enrichment of matching genetic ancestry at nuDNA-encoded mitochondrial genes (Zaidi and Makova, 2019), in order to minimize potential negative effects between mitochondrial proteins encoded in nuDNA and mtDNA. To look for such a signature, we classified nuclear-encoded mitochondrial genes into “high-mt” (*n* = 167) and “low-mt” (*n* = 793), which correspond to their importance to mitochondrial function as curated by Sloan et al. (2015) The rest of the nuclear genes were classified as “non-mt” (*n* = 17,456). We utilized an unweighted block bootstrap approach described in Zaidi and Makova (2019) to determine whether local ancestry in each gene category was significantly enriched or depleted compared to the global ancestry average of the population. Ancestry-informative SNPs were used to estimate genetic ancestry at 5-Mb segments overlapping each gene. For each gene category, we performed a random sampling with replacement (167 times to match the smallest gene category) and computed the deviation in mean local ancestry from the mean global ancestry. To compare the bootstrapped distributions of “high-mt” and “low-mt” genes to the “non-mt” genes, we utilized a *t*-test of independent means.

## Results

### Mitonuclear DNA Discordance in African Americans and European Americans

To investigate potential effects of mitonuclear DNA discordance on mtDNA gene expression, we required samples from an admixed population, for which both genotype and expression data are available. The GTEx dataset (GTEx Consortium, 2020) features a substantial number of genotyped samples (N = 101) from a recently admixed population, African Americans. Our global nuclear genetic ancestry analysis (see Methods) indicated that most genotyped African American individuals in GTEx hold a varying degree of African and European ancestry, with negligible contribution from other ancestral populations (Supplementary Figures S1, S3), consistent with prior studies (Parra et al., 1998; Tian et al., 2006; Bryc et al., 2015). Individuals from other populations with known recent admixed ancestry are present in the GTEx dataset in low numbers, which prevented us from including them in the analysis. For a comparison with African Americans, we utilized GTEx samples from a population with a limited recent admixture, European Americans. Our global nuclear genetic ancestry analysis of genotyped European Americans included in GTEx (N = 688) confirmed that the vast majority of them hold a predominantly European ancestry (Supplementary Figure S3).

To compute mitonuclear discordance, we first identified mitochondrial DNA haplogroups for the studied individuals (Table 1). Most African Americans studied (87%) carried the L haplogroup, thought to originate in Africa (van Oven and Kayser, 2009). The remaining African Americans studied carried the H, U, or T haplogroups, thought to originate in Europe or Eurasia (Richards et al., 1998; Loogväli et al., 2004; Fernandes et al., 2012; Fu et al., 2013). The vast majority of European Americans (99%) carried haplogroups thought to originate in Europe, with half of the individuals (50%) carrying the H haplogroup. A small proportion of European Americans (<1%) carried the African haplogroup L. As a result of this analysis, we determined whether each individual carried an mtDNA haplogroup of African or European/Eurasian ancestry.

**FIGURE 1:**
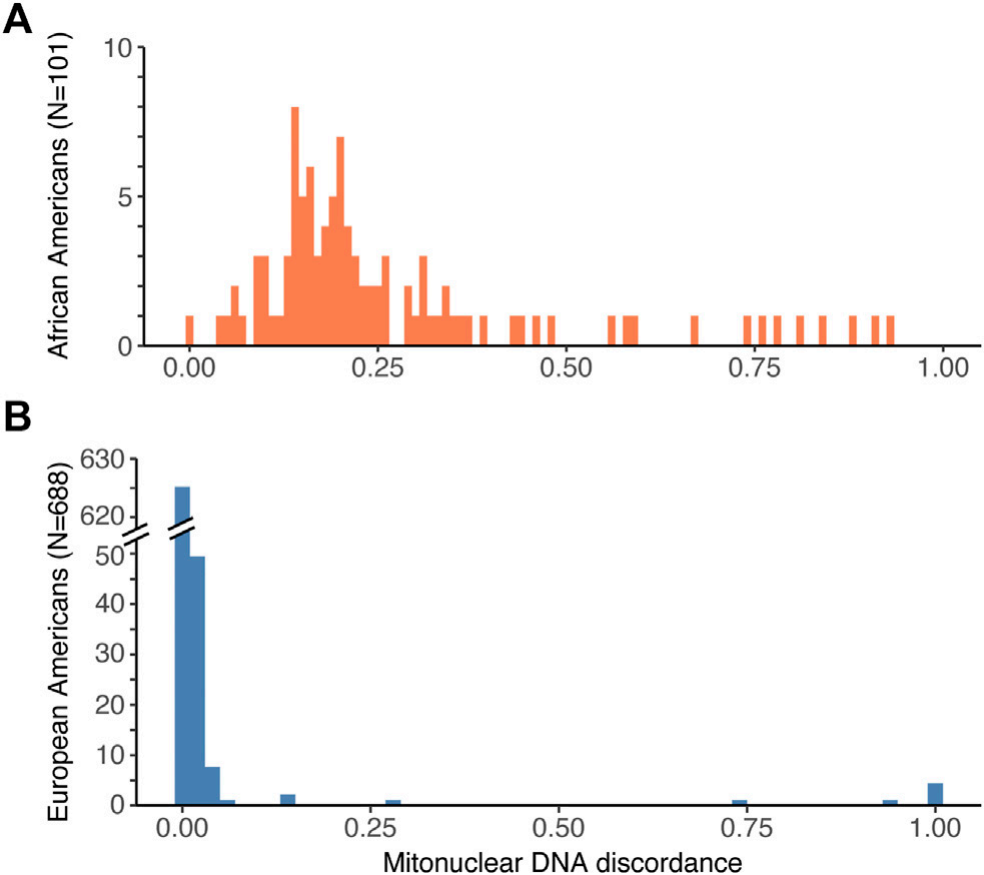
Histogram of mitonuclear DNA discordance values for **(A)** African Americans, and **(B)** European Americans. Note that the range of the *Y* axis is much smaller in **(A)** than in **(B)**, which reflects the smaller sample size for African Americans than for European Americans among the genotyped individuals in the GTEx dataset.

Second, we computed the fraction of the global nuclear genetic ancestry that does not match the mtDNA ancestry. This fraction corresponds to mitonuclear DNA discordance. For instance, for an African American individual with the African haplogroup L, mitonuclear DNA discordance is equal to the fraction of European global nuclear ancestry (e.g. 0.25). For African Americans (Figure 1A) we observed a wide spread of mitonuclear DNA discordance values (range from 0 to 0.928, median = 0.199, mean = 0.267, variance = 0.0421), reflecting the high variability in the proportion of African vs. European ancestry among these individuals (Supplementary Figure S3A). In contrast, the mitonuclear DNA discordance for European Americans (Figure 1B) was usually low (median = 9.99 × 10^–6^, mean = 0.0113), and, despite a handful of outliers with the African haplogroup L, exhibited a narrow spread (variance = 0.00799; Supplementary Figure S3B).

### Mitonuclear DNA Discordance and mtDNA Expression

The GTEx (Genotype-Tissue Expression) Consortium (GTEx Consortium, 2020) features a comprehensive collection of gene expression data across human tissues. The demand for energy production, and, consequently, dependence on optimal mitochondrial function, varies widely from tissue to tissue. Thus, if mitonuclear discordance impacts mitochondrial DNA gene expression, this would be more readily observed in energetically-demanding tissues. For this reason, we focused our analysis on mtDNA expression in skeletal muscle, heart muscle (ventricle and atrium), smooth muscle (esophagus and tibial artery), and nerve (tibial nerve, Table 2). We also included whole blood because this tissue had a high abundance of samples in the GTEx dataset. The gene expression data for these tissues in GTEx were available for a substantial number of genotyped African Americans and European Americans, ranging from 41 to 85, and from 311 to 580 individuals, respectively, per tissue (Table 2).

**TABLE 2:** The number of samples with gene expression data available per tissue, for genotyped individuals. The tissues are listed in the order of decreasing sample sizes in the overall dataset.

Tissue/Population	African Americans	European Americans	Total
Skeletal muscle	85	580	665
Whole blood	79	550	629
Smooth muscle (tibial artery)	75	471	546
Nerve (tibial)	66	431	497
Smooth muscle (esophagus)	64	435	499
Heart muscle (ventricle)	42	321	363
Heart muscle (atrium)	41	311	352

Prior to the analysis, gene expression levels were adjusted for age and ischemic time, and we subsequently call them “adjusted gene expression values” (see Methods for details). We also determined that adjusted expression levels were not significantly different between males and females for any tissue and gene analyzed, except for CO2 and ND4 in esophagus (Supplementary Table S2). Also, for the majority of genes analyzed, the particular Eurasian haplogroup did not have a significant effect on gene expression (Supplementary Tables S3, S4), and thus we did not include haplogroup designation in the subsequent analysis. The expression levels of ribosomal and transfer mitochondrial RNA, as well as of two protein-coding genes (*ND5* and *ND6*), were not analyzed because the GTEx RNA-Seq protocol enriches for poly(A)-containing RNA (GTEx Consortium, 2017) (see Methods for details). Indeed, *ND6* transcripts are not polyadenylated (Temperley et al., 2003; Temperley et al., 2010; Mercer et al., 2011; Kuznetsova et al., 2017) and even poly-A tails in *ND5* transcripts may only range from 0–10 bp (Temperley et al., 2010).

### The Levels of mtDNA Gene Expression are Lower in the Population With Higher Mitonuclear DNA Discordance

The machinery of mtDNA gene transcription is encoded by nuclear DNA, whereas the templates themselves, as well as the regulatory sequences (e.g., promoters), are encoded by mtDNA. Therefore we can expect discordance between these genomes to affect the levels of mtDNA gene expression. Specifically, we hypothesized that higher mitonuclear DNA discordance corresponds to lower mtDNA gene expression.

**FIGURE 2:**
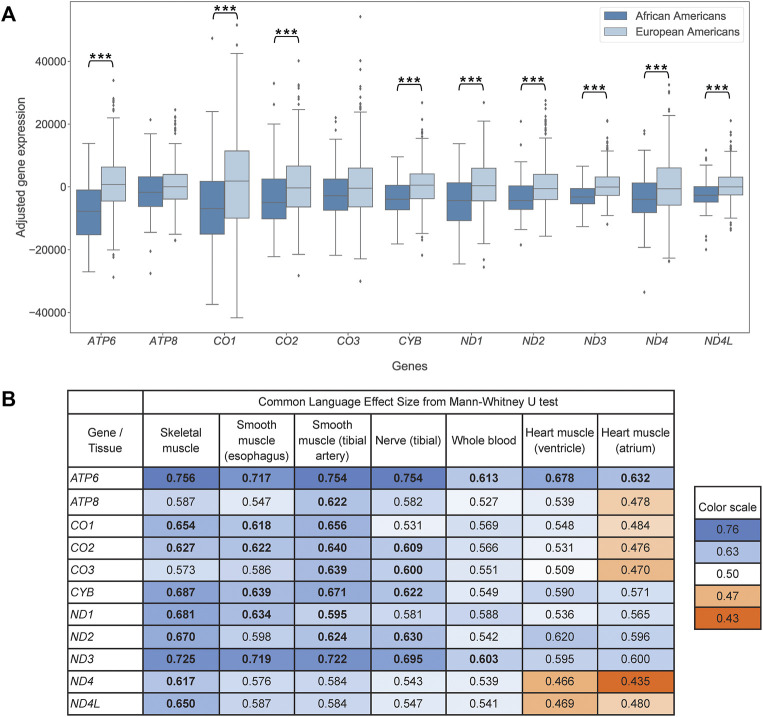
**(A)** Pairwise boxplot of skeletal muscle mtDNA gene expression in African Americans (*n* = 85) and European Americans (*n* = 580), adjusted by age and ischemic time. Stars indicate significant results (*p* < 0.05) of one-sided, Bonferroni-corrected Mann-Whitney U test of mean ranks (* indicates *p* < 0.05, ** indicates *p* < 0.01, *** indicates *p* < 0.001). **(B)** Mann-Whitney U test common-language effect sizes comparing adjusted mtDNA gene expression between African Americans and European Americans for skeletal muscle, smooth muscle (esophagus), artery (tibial), nerve (tibial), whole blood, and heart (ventricle and atrium). Bold values indicate significant difference (*p* < 0.05) using a one-sided, Bonferroni-corrected Mann-Whitney U test of mean ranks. p-values are listed in Supplementary Table S5. Values on the blue background, above 0.5, support lower adjusted expression with higher mitonuclear DNA discordance, consistent with our hypothesis; values on the orange background, below 0.5, support higher adjusted expression with higher mitonuclear DNA discordance. The background is darker with increasing effect. Bonferroni correction accounts for multiple hypothesis testing across the 11 mtDNA protein-coding genes analyzed.

To test this hypothesis, we compared protein-coding mtDNA gene expression between African Americans and European Americans, the populations with on average high and low mitonuclear discordance, respectively. In skeletal muscle, the tissue with the largest sample size in our data set, we observed lower adjusted expression levels for African Americans than for European Americans for 11 protein-coding genes analyzed, consistent with our hypothesis (Figure 2A). This was significant for nine genes (one-sided Mann-Whitney U test, see Supplementary Table S5 for Bonferroni-corrected *p*-values). The results did not change qualitatively if, for a random subset of data, we considered genes from two bicistronic transcripts together (Supplementary Figure S4).

Tibial artery, tibial nerve, esophagus, and whole blood revealed a pattern similar to that observed above for skeletal muscle. In these four tissues, the same 11 protein-coding mtDNA genes exhibited lower adjusted expression levels for African Americans than for European Americans (with significant *p*-values for nine, six, six, and two genes, respectively; Figure 2B, Supplementary Figure S5, Supplementary Table S5). Heart ventricle had nine genes following this pattern (one comparison was significant, *ATP6*). Heart atrium, the tissue with the smallest sample sizes in our data set, had five genes with lower adjusted expression levels for African Americans than for Europeans (one comparison was significant, *ATP6*).

### MtDNA Gene Expression Levels are Usually Lower in Individuals With High Mitonuclear DNA Discordance

Among genotyped individuals in GTEx, some African Americans had low mitonuclear DNA discordance and some European Americans had high mitonuclear DNA discordance. Therefore, in a separate analysis, we performed a permutation test that compares mean adjusted expression levels for each mtDNA protein-coding gene and each tissue between individuals with high vs. low mitonuclear discordance, independent of the population to which these individuals belong to. We used the mean mitonuclear discordance across genotyped African Americans and European Americans in each tissue, to classify individuals as the ones having low (below the mean) vs. high (above the mean) discordance. Next, in each permutation, we randomly assigned individuals to either the low- or the high-discordance group, keeping the sample sizes of these two groups consistent with those in the original dataset, and computed the difference in the mean adjusted gene expression between low- and high-discordance groups. This was performed 1,000 times. We then compared the resulting distribution of the differences in the mean adjusted gene expression with the observed one. Similar to the logic in the previous section, we hypothesized that higher mitonuclear discordance leads to lower mtDNA gene expression.

**FIGURE 3:**
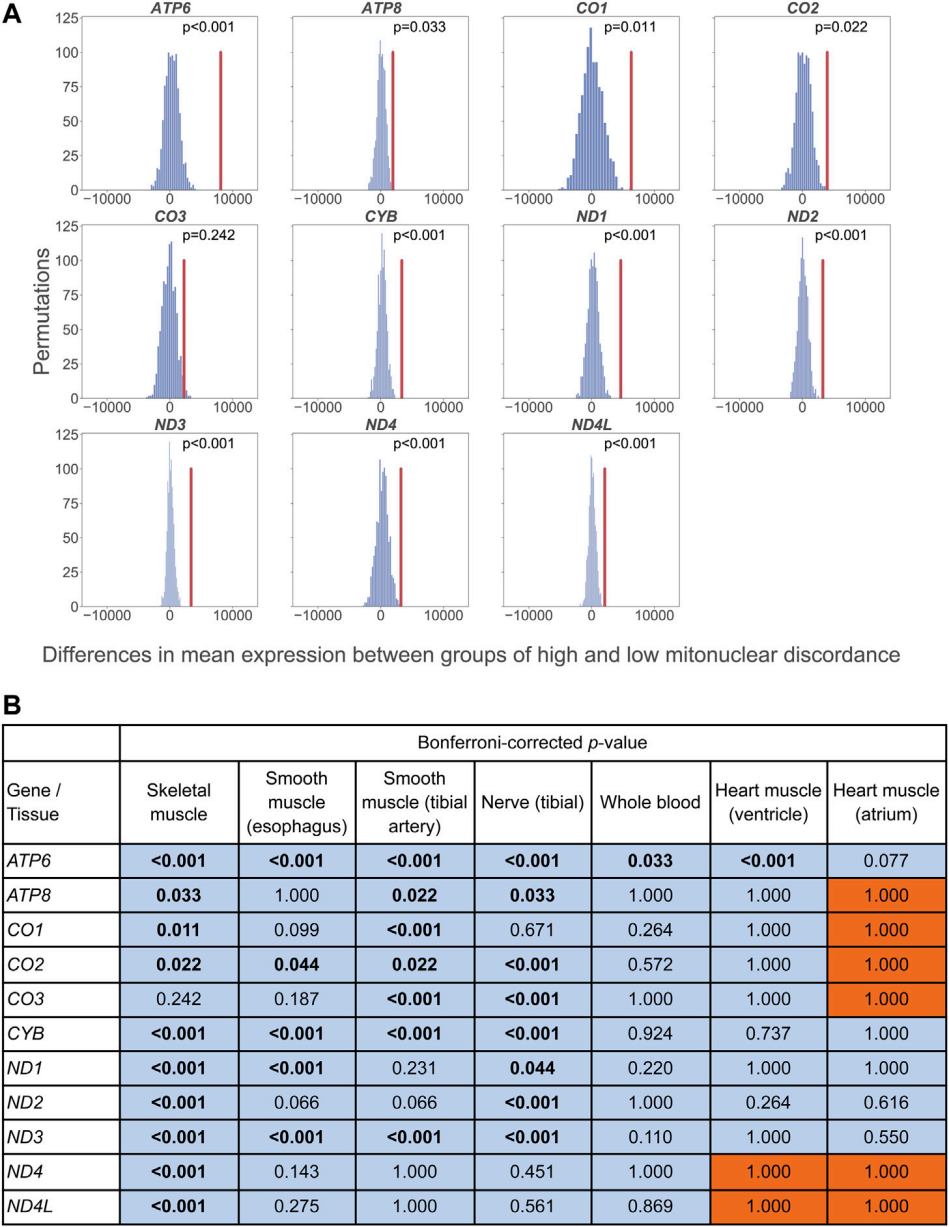
**(A)** Permutation test comparing the mean gene expression (adjusted by age and ischemic time) for skeletal muscle in high vs. low mitonuclear DNA discordance groups, regardless of which population each individual belongs to. The distribution is the expected differences in mean adjusted gene expression between groups (with permuted labels). These groups were determined using a cutoff at the mean mitonuclear DNA discordance. The vertical red line indicates the observed difference in mean TPM between groups in our dataset. **(B)** Bonferroni-corrected, one-sided permutation test results for skeletal muscle, smooth muscle (esophagus), artery (tibial), nerve (tibial), whole blood, and heart (ventricle and atrium). The mean mitonuclear discordance values were used as the threshold for high and low discordance groups, for each tissue (0.042, 0.047, 0.048, 0.045, 0.045, 0.043, and 0.033, respectively). Bold text indicates significant results (*p* < 0.05). Values on the blue background indicate lower adjusted expression with higher mitonuclear DNA discordance, consistent with our hypothesis; values on the orange background indicate higher adjusted expression with higher mitonuclear DNA discordance.

Consistent with our hypothesis, for skeletal muscle, in all 11 mtDNA genes analyzed, the decrease in adjusted expression levels in the high-discordance vs. the low-discordance group was greater than expected by chance alone (significant for ten genes; Figure 3A). The permutation analysis in tibial artery, tibial nerve, esophagus, and whole blood indicated a similar pattern for the same 11 genes as that observed for skeletal muscle, with significant *p*-values for seven, eight, five, and one gene, respectively (Figure 3B). The trends for heart ventricle were consistent with our hypothesis for nine out of 11 genes analyzed, with one significant *p*-value (*ATP6*). For heart atrium, trends consistent with our hypothesis were observed for five out of 11 genes analyzed, with no significant tests. Note that, because this tissue has the smallest sample size in our data set, we had limited power to detect differences in gene expression.

### A Trend Towards a Negative Correlation Between Mitonuclear DNA Discordance and mtDNA Expression Levels

**FIGURE 4:**
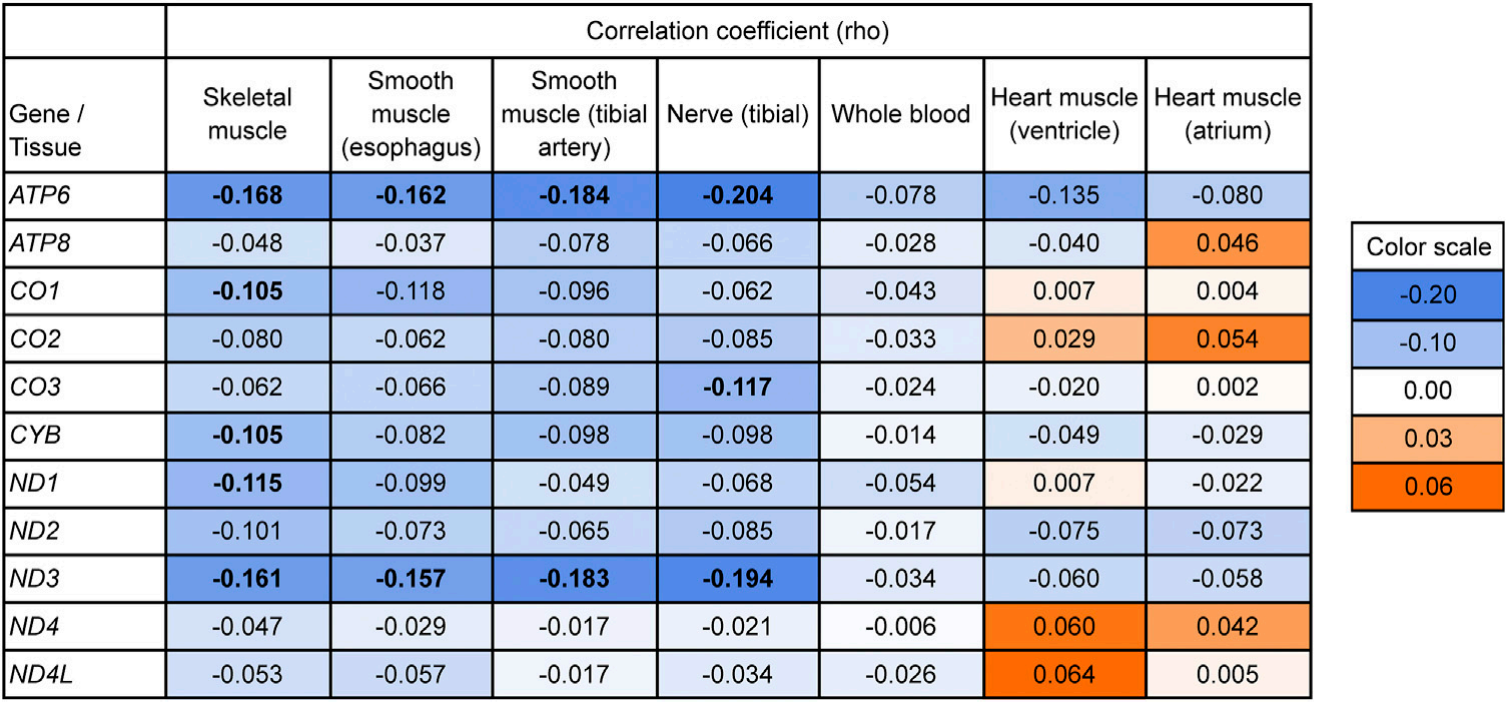
Testing the relationship between mitonuclear DNA discordance and mtDNA gene expression (adjusted for age and ischemic time) using a one-sided Spearman’s rank-order correlation. Bold values indicate Bonferroni-corrected significant results (*p* < 0.05). *p*-values are listed in Supplementary Table S3. Values on the blue background indicate lower adjusted expression with higher mitonuclear DNA discordance, consistent with our hypothesis; values on the orange background indicate higher adjusted expression with higher mitonuclear DNA discordance. The background is darker with increasing effect. Bonferroni correction accounts for multiple hypothesis testing across the 11 mtDNA protein-coding genes analyzed.

To directly test for a relationship between mitonuclear discordance and adjusted mtDNA gene expression levels, we performed a correlation analysis including individuals from both populations. If our hypothesis is correct, we expect a negative correlation between these two variables. Note that the distribution of mitonuclear DNA discordance values in our dataset is skewed towards small values because of low discordance in European Americans (Figure 1). Therefore, for each tissue and gene analyzed, we computed a Spearman’s rank-order correlation coefficient between adjusted mtDNA gene expression and mitonuclear DNA discordance. In skeletal muscle, Spearman’s correlation coefficient was negative, albeit low, for 11 genes, with five genes (*ATP6*, *CO1*, *CYB*, *ND1*, and *ND3*) having significant negative correlations, consistent with our hypothesis (Figure 4).

Mitonuclear discordance and adjusted gene expression levels exhibited a trend towards a negative correlation for most mtDNA genes analyzed for esophagus, tibial artery, and tibial nerve, with two genes, *ATP6* and *ND3*, showing significant correlations (Figure 4, see Supplementary Table S6 for Bonferroni-corrected *p*-values). The results for whole blood followed the same trend, but did not have significant correlations. The results for heart ventricle and heart atrium were mixed.

### Ancestry at Nuclear-Encoded Mitochondrial Genes in African Americans

**FIGURE 5:**
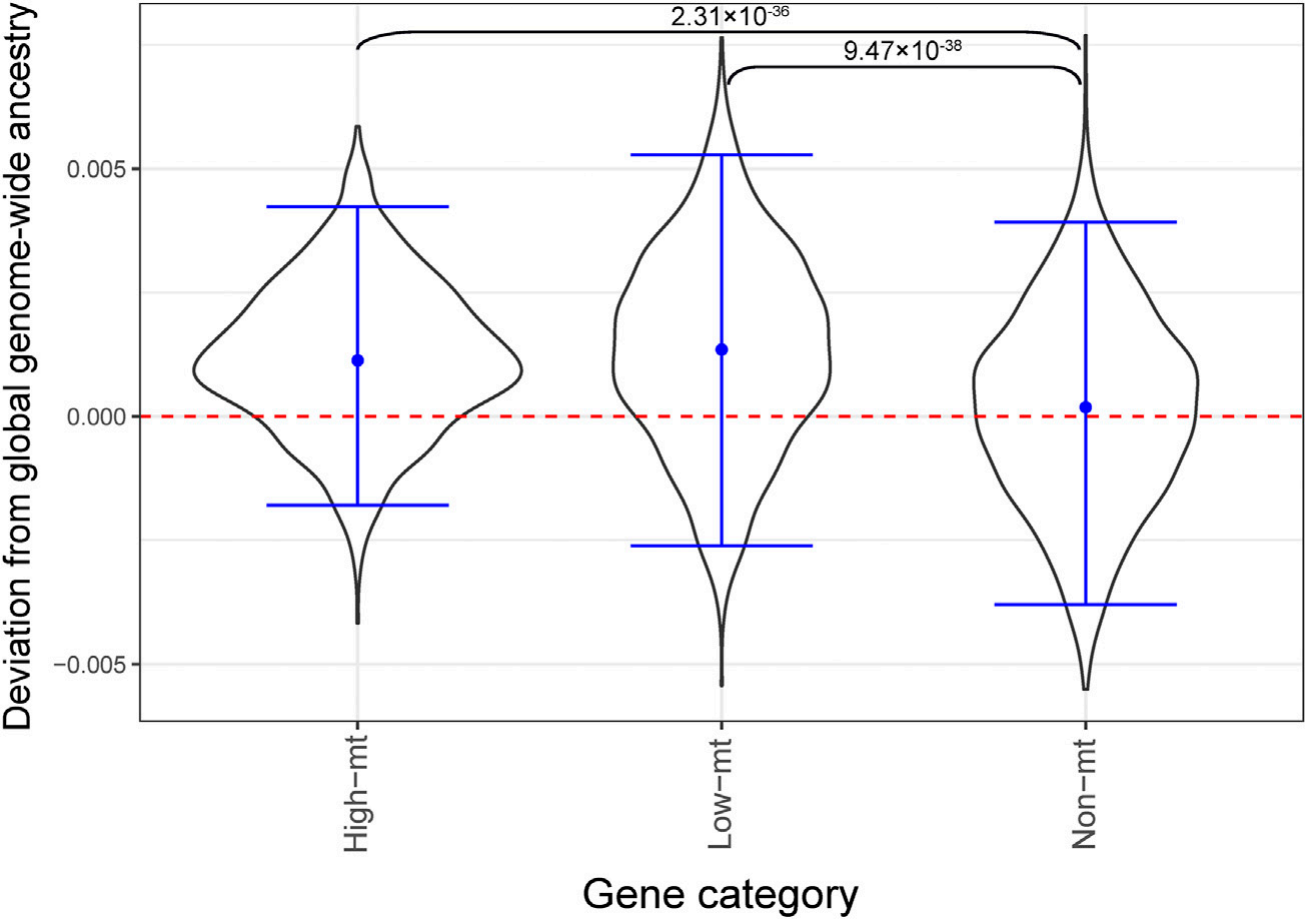
Local ancestry enrichment for African ancestry in African Americans from the GTEx dataset. The *Y* axis shows local ancestry deviation and the *X* axis shows functional categories (high-mt: 167 genes, low-mt: 793, non-mt: 17,456 genes, see text for details). A block bootstrap approach (see Methods) was used to generate the distributions of deviation from global ancestry. Blue horizontal bars show the empirical 95% CI of the mean ancestry deviation. The *p*-values correspond to a *t*-test of independent means comparing mean local ancestry deviations at non-mt genes to those at high-mt and low-mt genes.

If mitonuclear DNA discordance has an effect on mitochondrial function, selection might act to reduce mitonuclear discordance in the population by favoring the same ancestry as the mtDNA at nuclear-encoded mitochondrial genes. To test this hypothesis, we studied deviations of local ancestry at nuclear-encoded mitochondrial genes (see Methods for details) from the mean global, genome-wide ancestry, for African Americans (Figure 5). If our hypothesis is correct, we expect to observe an enrichment in African ancestry for nuclear-encoded mitochondrial genes for African Americans, because the vast majority of them possess the African haplogroup L. Two groups of nuclear-encoded mitochondrial genes were considered (Sloan et al., 2015)—“high-mt” genes and “low-mt” genes, with the first group including 167 high-confidence mitochondrial genes (encoding proteins that are part of the mtDNA replication and transcription machinery, and of ribosomal and OXPHOS complexes) and the second group including 793 genes whose products are known to be imported to the mitochondrion (Pagliarini et al., 2008). The remaining 17,456 genes were called “non-mt.” In African Americans, the 95% bootstrapped distributions of deviations from the mean global genome-wide ancestry overlapped, and thus were not significantly different, among “high-mt,” “low-mt,” and “non-mt” genes (Figure 5). However, consistent with our expectations, we did observe a significantly higher mean African ancestry for “high-mt” than “non-mt,” as well as for “low-mt” than “non-mt,” genes in African Americans (*p* = 2.31 × 10^–36^ and *p* = 9.47 × 10^–38^, respectively, two-sided *t*-test of independent means; Figure 5).

## Discussion

In this paper, we investigated whether mitonuclear discordance in genetic ancestry is associated with mtDNA gene expression in human populations. Utilizing GTEx samples from a recently admixed human population, African Americans, and a population that likely experienced only limited recent admixture, European Americans, we found support of our hypothesis of decreased mtDNA gene expression with higher mitonuclear discordance, as evaluated by three different analyses performed in six energetically demanding tissues and in whole blood. First, we observed a trend towards lower adjusted gene expression in the *population* with higher mitonuclear discordance for the majority of mtDNA protein-coding genes. Second, by contrasting adjusted gene expression and mitonuclear DNA discordance, disregarding population, we noticed a consistently lower adjusted mtDNA gene expression in *individuals* with higher mitonuclear discordance for the majority of mtDNA protein-coding genes. Third, the majority of mtDNA genes exhibited negative correlations with mitonuclear DNA discordance. The observed trends were the strongest in skeletal muscle, the tissue for which we had the largest sample size. For the other tissues analyzed, the number of significant tests decreased with the sample size. Nevertheless, in all seven tissues analyzed, we observed a consistent trend towards lower adjusted gene expression at higher mitonuclear DNA discordance for the mtDNA protein-coding genes.

Despite this observation, the differences in adjusted gene expression levels between the two populations studied were at most 26% in magnitude (Supplementary Table S7), with the median difference across genes and tissues of only 9% (Supplementary Table S7). The relatively small differences in adjusted mtDNA expression values as related to nuclear genetic background require a further investigation of the strength of their physiological effects. A natural extension of our work would be conducting an experimental, functional analysis of mitochondrial DNA discordance in human cell lines, for instance, to directly assess mitochondrial respiration. Ideally, these cell lines would be established from a recently admixed population with a high variance in mitonuclear DNA discordance. A direct assessment of mitochondrial function at varying levels of mitonuclear DNA discordance has not been performed in a human population. It is possible that the observed differences in adjusted gene expression levels can be explained in part by differences in mtDNA copy number, a mitochondrial phenotype also found to be related to mitonuclear discordance (Zaidi and Makova, 2019; Yang et al., 2021). This possibility will have to be explored in data sets containing energetically demanding tissues for which both mtDNA copy number and expression levels are evaluated, which is not the case for recently admixed populations in GTEx.

### Potential Selection for Matching Mitochondrial and Nuclear Ancestry

Next, we tested whether there is an enrichment for African ancestry at nuclear-encoded mitochondrial genes (relative to the genomic background) in African Americans. If there are indeed incompatibilities between mtDNA and the nuclear genome, we would expect selection to favor African ancestry at nuDNA-encoded mitochondrial genes as the mtDNA in this population predominantly harbors the L haplogroup found in Africa. In African Americans, forced migrations (Mathias et al., 2016) led to an initial admixture event approximately six generations ago (Bryc et al., 2015). During that time, selection might have acted on nuDNA sites to match the prevalent mtDNA ancestry. For African Americans in our sample, we did observe a significantly higher average African ancestry at nuDNA-encoded mitochondrial genes (“high-mt” or “low-mt”) than at non-mitochondrial genes, even though the underlying bootstrapped distributions from global ancestry overlapped zero, suggesting a relatively weak signal. Zaidi and Makova (2019) observed a similar yet stronger signal (bootstrapped distribution of deviations from global ancestry did not overlap zero) of matching ancestry at nuDNA-encoded mitochondrial genes in the 1000 Genomes’ African American population (ASW). In fact, out of the six admixed populations studied by Zaidi and Makova (2019) from the 1000 Genomes Project, two (African Americans and Puerto Ricans) had a significant enrichment in matching ancestry, whereas one (Mexican Americans) had an unexpected enrichment in a mismatching ancestry, at nuDNA-encoded mitochondrial genes. These and our present results suggest that selection is acting to preserve a matching ancestry at sites important for mitochondrial function to reduce mitonuclear incompatibility. However, since this previous study indicated population-dependent signatures in matching mitochondrial and nuclear ancestries (Zaidi and Makova, 2019), it will be important to study gene expression in other recently admixed populations. Moreover, given this enrichment for the matching ancestry observed in our study, it would be interesting to experimentally test whether nuDNA-encoded protein subunits of different ancestries affect mtDNA transcriptional complexes.

### *ATP6* and *ND3*

Whereas the trend of decreasing adjusted gene expression levels with increasing mitonuclear incompatibility was found for the majority of genes in most tissues studied, two mtDNA genes in particular—*ATP6* and *ND3*—showed consistently significant signals across analyses and across tissues. *ATP6* displayed the highest magnitude effects in all three analyses conducted across all tissues. *ATP6* encodes a key component of the ATP synthase (Complex V) (Zhao et al., 2019). A previous study demonstrated that expression levels of this gene are directly correlated with ATP levels in the mitochondria (Amiott and Jaehning, 2006). *ATP6* is an essential component of Complex V and ATP synthesis. A subset of Complex V deficiencies are caused by mutations in *ATP6* (Schon et al., 2001), and may lead to severe neural impairment in NARP (neuropathy, ataxia, and retinitis pigmentosa) and maternally inherited Leigh syndrome. Importantly, there is evidence that variation in nuDNA-encoded mitochondrial genes also produces deficiencies in ATP synthase function and leads to clinical symptoms (Sperl et al., 2006). This suggests that mitonuclear interactions may also play an effect in the presentation of these clinical cases where *ATP6* is implicated.

*ND3* had the second highest magnitude effects in all three analyses conducted across all tissues. *ND3* encodes NADH dehydrogenase, a subunit of Complex I, the largest complex in the electron transport chain (Zhao et al., 2019). *ND3* handles electron transport in Complex I alongside the products from *ND1*, *ND2*, and *ND4-6* genes. Therefore, it is surprising that only *ND3* gene expression is so strongly correlated with mitonuclear discordance. However, Complex I switches between catalytically activate (A) and deactivate (D) states (Burger et al., 2021), and exposure of the Cys39 residue in the ND3 subunit is a defining feature in this switch. If the residue is modified, it blocks the reactivation of Complex I and limits NADH oxidation (Burger et al., 2021). This could explain how *ND3* stands out from the other genes forming Complex I. Thus, both *ATP6* and *ND3* encode critical components of the electron transport chain, and alterations in their gene expression might affect this fine-tuned system. Our results suggest that these two genes are affected by mitonuclear ancestry discordance in the present dataset the most.

Noteworthy, Cohen et al. (2016) observed lower gene expression levels for several mtDNA protein-coding genes, including *ATP6* and *ND3,* in presumably unadmixed individuals with the African L haplogroup (in Yorubans) than in those with non-African haplogroups (Finns and Italians) from the 1000 Genomes Project (in samples derived from lymphoblastoid cell lines). They suggested that the L haplogroup may have a lower gene expression regardless of nuclear background. Because the majority of African Americans in our data set had the L haplogroup, we cannot exclude a possibility that the associations we observed are due to specific gene expression signatures associated with the L haplogroup. In the 1000 Genomes dataset, we observed 8 and 6 inter-promoter region DNA variants specific to, and shared by, individuals with L and H haplogroups, respectively. Variation in this critical regulatory region may contribute to the observed differences in mtDNA expression. However, the study by Cohen et al. (2016) examined only one cell line, which has comparatively low energetic demands, as compared with multiple energetically demanding tissues we studied here. Therefore, we cannot compare their results directly to ours. However, if mitonuclear discordance did not contribute to our results, we would not have observed a negative *correlation* between mtDNA expression and mitonuclear discordance. Ideally, one would like to test whether the presence or absence of mitonuclear discordance influences gene expression levels for the same haplogroup. We attempted such an analysis (Supplementary Figure S6, Supplementary Table S8), however, were severely limited by the sample sizes for African Americans with European haplogroups and for European Americans with African haplogroups, and could not obtain meaningful results.

### Potential Biomedical Implications

Mitonuclear DNA discordance may affect admixed individuals at different levels—mitochondrial, cellular, tissue, organismal, and populational. Here we show that mitonuclear DNA discordance is linked to adjusted gene expression levels of mtDNA-encoded protein-coding genes, and our previous study demonstrated its effects on mtDNA copy number (Zaidi and Makova, 2019). Note that both effects were relatively small in magnitude. Nevertheless, the biomedical implications of mitonuclear DNA discordance on admixed populations should be considered due to their potential adverse effects. For instance, it was suggested that divergent nuclear and mitochondrial ancestry increases the risk of preterm birth (Crawford et al., 2018). However, mitonuclear discordance is only one of many factors that need to be taken into account when evaluating individual health. For instance, heterosis, a greater fitness in the progeny than in both parents (Birchler et al., 2010), would be a positive effect resulting from admixture. Given that mitochondrial function is a complex trait, we expect multiple related factors to contribute to the overall fitness of admixed individuals. Studies in admixed populations also shed light on the consequences of mitonuclear DNA discordance for mitochondrial replacement therapy. Recently it was suggested that such discordance is not expected to be harmful to individuals due to potential deleterious effects of only small effect sizes (Eyre-Walker, 2017). Additional investigation of mitochondrial phenotypes in admixed populations and individuals with mitochondrial replacement therapy is warranted particularly because of recent studies demonstrating the sizable effect of mitochondrial variation on complex traits in humans (Yonova-Doing et al., 2021).

### Lack of Data for Diverse Populations

A limitation of this work lies in the fact that we studied the effects of mitonuclear DNA discordance in only one recently admixed human population, African Americans, due to the small sample sizes for other admixed populations in the GTEx data set. We expect the gene expression trends we observed to vary among admixed populations depending on the levels of mitonuclear DNA discordance, which in turn depends on the levels of admixture. In addition, the sample size of African Americans with available genotypes in GTEx is notably smaller (*n* = 101) than that of European Americans (*n* = 688). The 1000 Genomes Project generated gene expression data from lymphoblastoid cell lines (analyzed by (Cohen et al., 2016)), however these were limited to European American (CEU), Italian (TSI), British (GBR), Finnish (FIN), and Yoruban (YRI) populations, which had experienced a more ancient admixture. These facts reflect the urgent need to include larger numbers of diverse populations, as well as larger sample sizes for such populations, in future genetic and gene expression studies. This lack of diversity in genetics research is well documented (Shringarpure et al., 2016; Bentley et al., 2017). We acknowledge the complex issue of sampling from historically marginalized groups, and that inclusion should be more equitable in order to achieve a more diverse scientific community, which in turn would improve how these populations are studied (Claw et al., 2018).

## Data Availability

Publicly available datasets were analyzed in this study. This data can be found in dbGaP at http://www.ncbi.nlm.nih.gov/gap through dbGaP accession number phs000424.v8.p2. Code is available at, https://github.com/makovalab-psu/mitonuclear_expression.
