# Supplementary material for: Exploring the Effects of Mitonuclear Interactions on Mitochondrial DNA Gene Expression in Humans
Source: Front Genet. 2022 Jun 29;13:797129. doi: 10.3389/fgene.2022.797129 (PMC9277102; doi:10.3389/fgene.2022.797129)

Supplementary figures

Figure S1. ADMIXTURE cross validation error for GTEx samples at K of 1, 2, 3, 4, and 5.

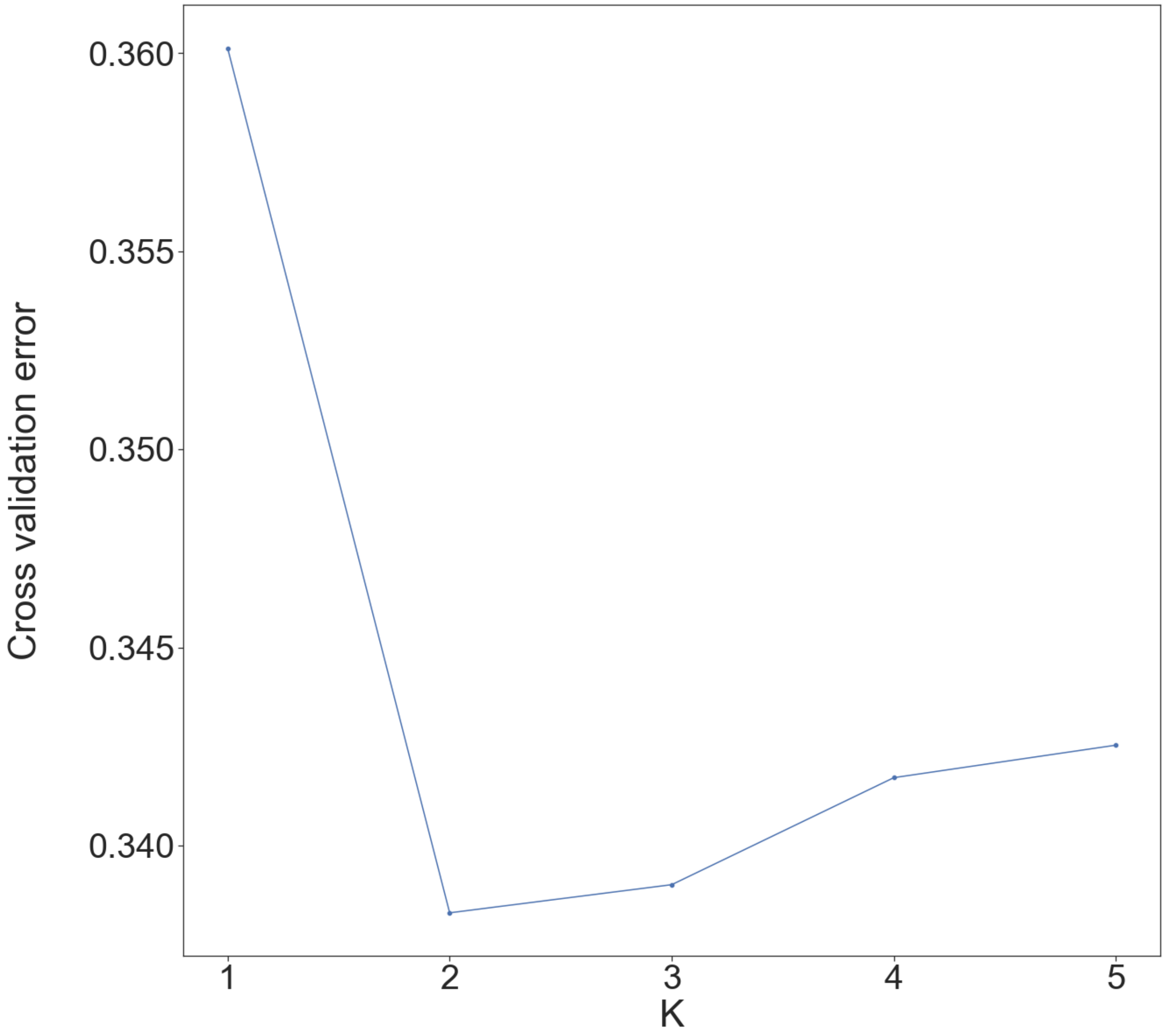

Figure S2. Global diploid ancestry estimated from local ancestry (RFMix) and global ancestry estimated from ADMIXTURE have high levels of correlation (Pearson correlation: European ancestry, R^2 = 0.999, *p*-value = 0.0; African ancestry, R^2 = 0.999, *p*-value = 0.0). The red line indicates *y*=*x*.

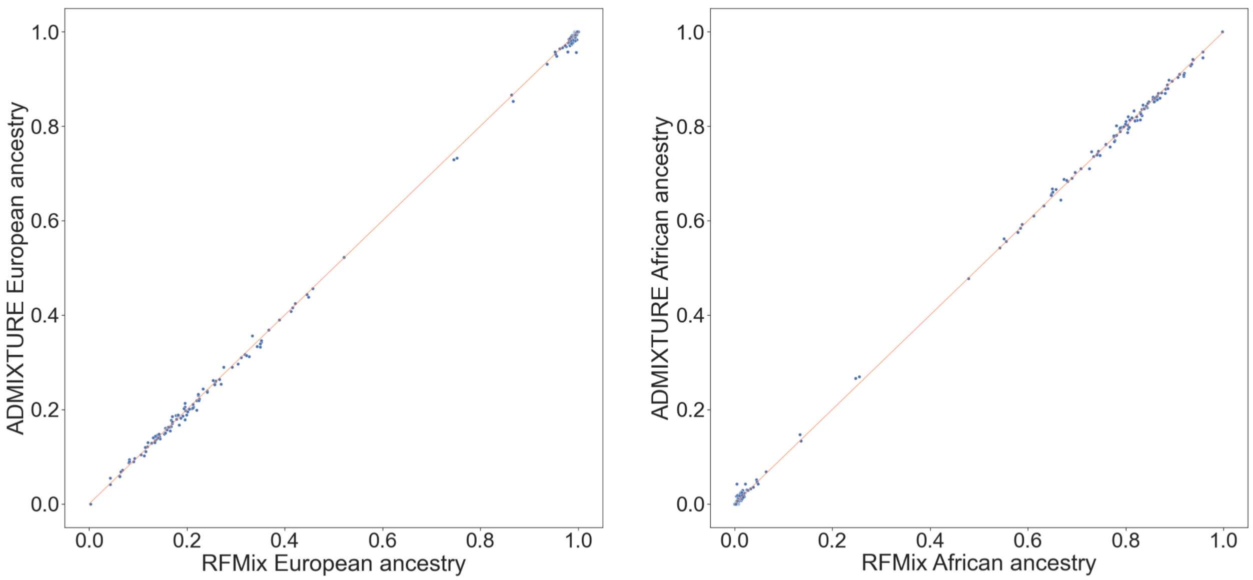

Figure S3. Global ancestry estimates for African Americans (**A**) and European Americans (**B**) in GTEx, estimated using ADMIXTURE. Each vertical line indicates a single individual. Red indicates African ancestry and blue indicates European ancestry.

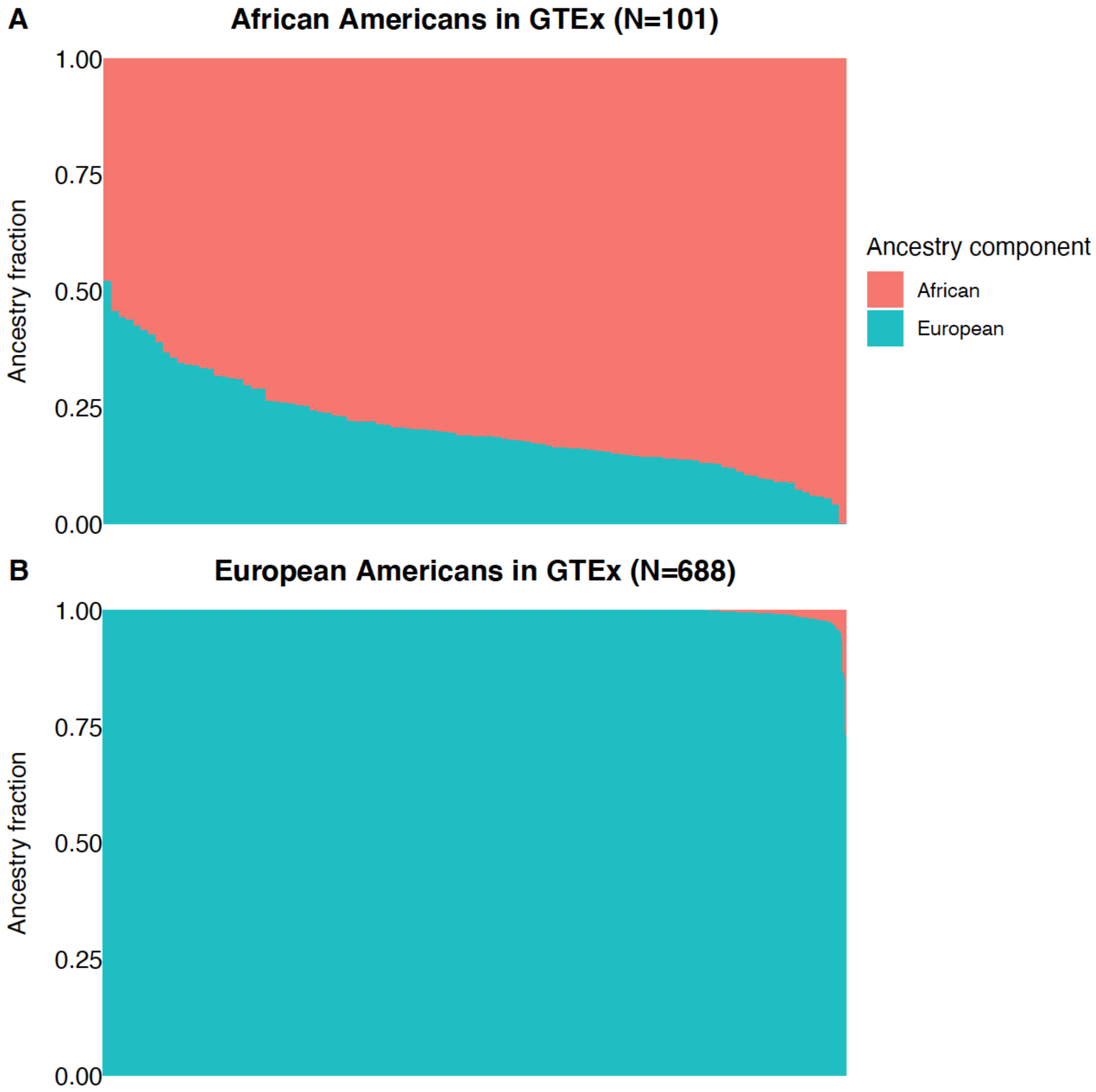

Figure S4. (A) Pairwise boxplots of expression in a reanalysis of RNA-Seq mapping to bicistronic transcripts (in the case of *ATP8/ATP6* and *ND4L/ND4*). These are plotted in normalized gene expression (TPM) as we did not find features (e.g. sex, age, ischemic time) to be correlated in this subset. **(B)** P-values of the Mann-Whitney U-test are shown below.

A

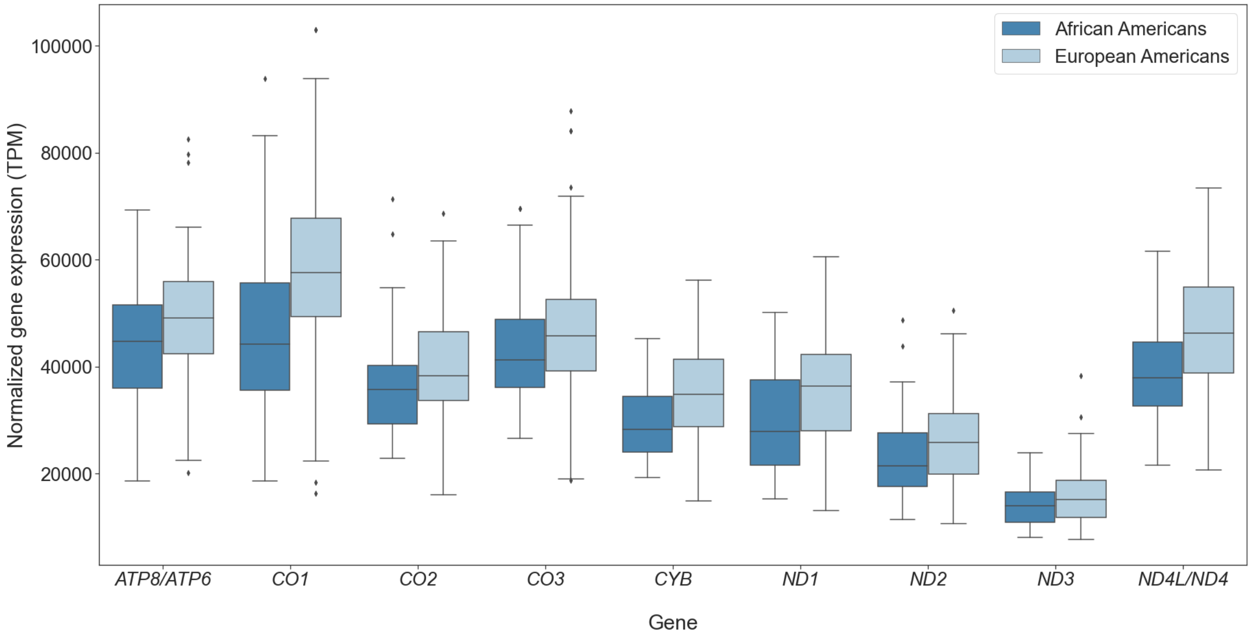

B

Mann-Whitney U test P-values			
**Gene**	**Statistic**	**P-value**	**Bonferroni-corrected P-value**
ATP8/ATP6	3837.5	**0.000507**	**0.005578**
ND4L/ND4	4054.5	**0.000025**	**0.00027**
ND1	4018.5	**0.000042**	**0.000464**
ND2	3736.5	**0.001714**	**0.018851**
ND3	3691.5	**0.002836**	**0.031199**
ND5	2491.5	0.934656	10.281216
ND6	2192.5	0.99502	10.94522
CO1	4076.5	**0.000017**	**0.000192**
CO2	3522.5	**0.015243**	0.167673
CO3	3452.5	**0.027798**	0.305774
CYB	4054.5	**0.000025**	**0.00027**

Figure S5. Pairwise boxplots of adjusted mtDNA gene expression compared across African Americans and European Americans. The sample sizes for each population are indicated. Stars indicate significant results (*p*<0.05) of one-sided, Bonferroni-corrected *t*-test of independent means.

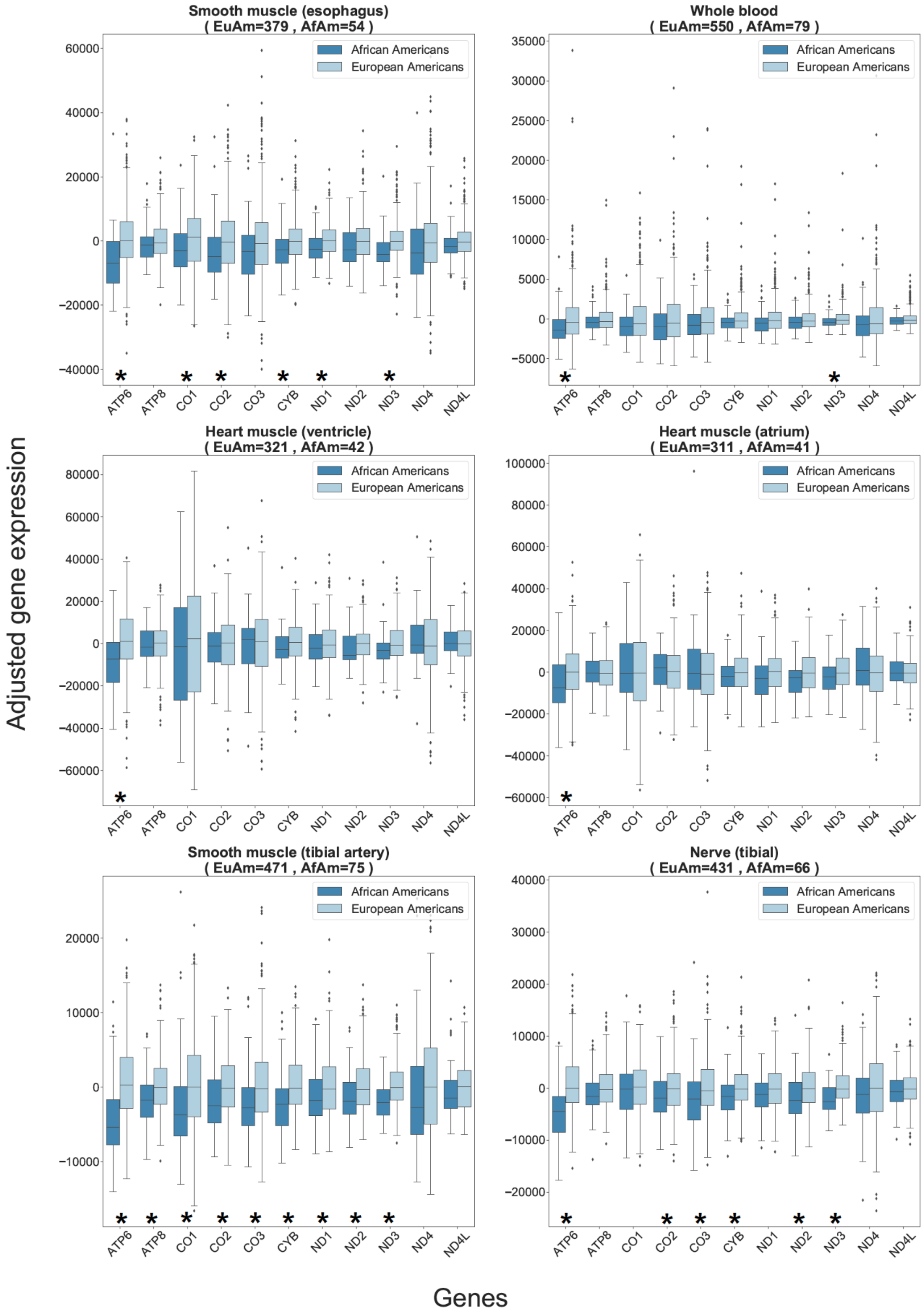

Figure S6. Pairwise boxplot of skeletal muscle adjusted mtDNA gene expression for the L mtDNA haplogroup in African Americans (N=74) and European Americans (N=4), as well as for the H mtDNA haplogroup in African Americans (N=7) and European Americans (N=291). See Table S8 for the results of Mann-Whitney U test.

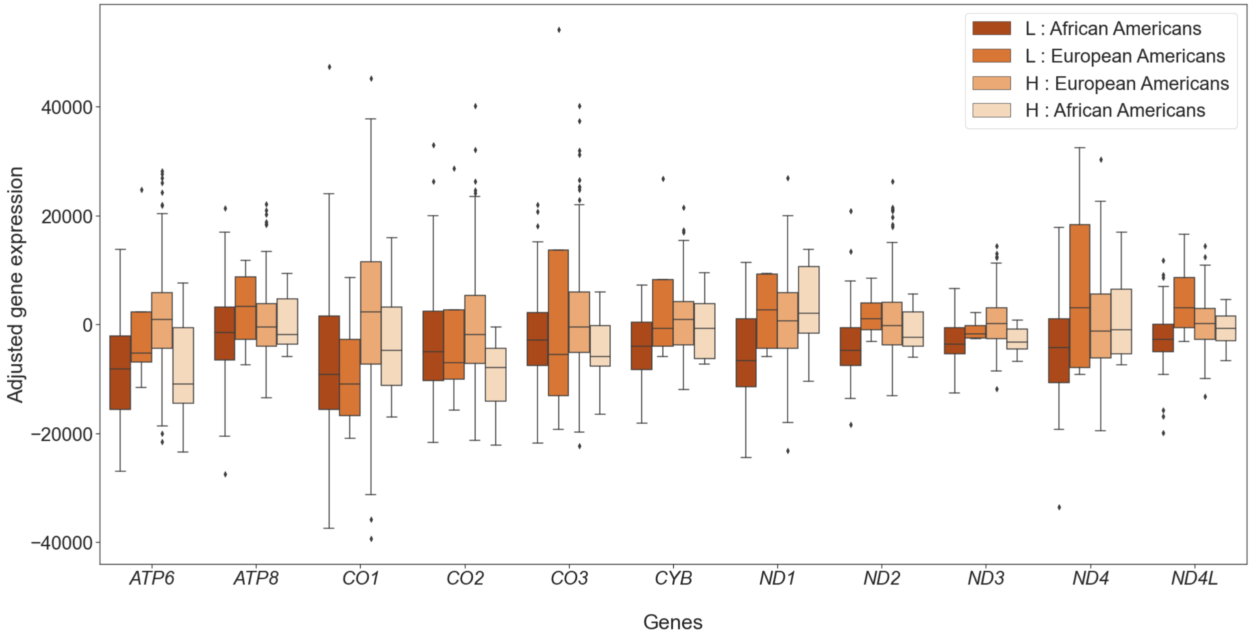

Supplement: Supplementary file 1 [file DataSheet1.pdf]
